# Impact of Co-Delivery of EGCG and Tuna Oil within a Broccoli Matrix on Human Gut Microbiota, Phenolic Metabolites and Short Chain Fatty Acids In Vitro

**DOI:** 10.3390/molecules27030656

**Published:** 2022-01-20

**Authors:** Meng Shi, Emma Watson, Michael Conlon, Luz Sanguansri, Mary Ann Augustin

**Affiliations:** 1 College of Food Science and Technology, Hunan Agricultural University, Changsha 410128, China; shimeng@hunau.edu.cn; 2 Tea Research Institute, Zhejiang University, Hangzhou 310058, China; 3 CSIRO Agriculture and Food, 671 Sneydes Road, Werribee, VIC 3030, Australia; luz.sanguansri@gmail.com (L.S.); maryann.augustin@csiro.au (M.A.A.); 4 CSIRO Health and Biosecurity, Kintore Ave., Adelaide, SA 5000, Australia; emma.j.watson80@gmail.com

**Keywords:** broccoli by-products, tuna oil, EGCG metabolites, short-chain fatty acid, gut microbe

## Abstract

(-)-Epigallocatechin gallate (EGCG) and tuna oil (TO) are beneficial bioactive compounds. EGCG, TO or a combination of, delivered by broccoli by-products (BBP), were added to an in vitro anaerobic fermentation system containing human fecal inocula to examine their ability to generate short-chain fatty acids (SCFA), metabolize EGCG and change the gut microbiota population (assessed by 16 S gene sequencing). Following 24 h fermentation, EGCG was hydrolyzed to (-)-epigallocatechin and gallic acid. EGCG significantly inhibited the production of SCFA (*p* < 0.05). Total SCFA in facal slurries with BBP or TO-BBP (48–49 µmol/mL) were significantly higher (*p* < 0.05) than the negative control with cellulose (21 µmol/mL). EGCG-BBP and TO-EGCG-BBP treatment increased the relative abundance of *Gluconacetobacter*, *Klebsiella* and *Trabulsiella*. BBP and TO-BBP showed the greatest potential for improving gut health with the growth promotion of high butyrate producers, including *Collinsella aerofaciens*, *Bacillus coagulans* and *Lactobacillus reuteri*.

## 1. Introduction

Macronutrients (e.g., carbohydrates, especially dietary fibre, protein and fats) and phytonutrients (e.g., polyphenols) have a role in shaping the composition of the gut microbiota, and this is highly associated with human health. Broccoli is a natural and rich source of carbohydrates, dietary fibre, protein and phytonutrients. Broccoli alters cecum microbiota and produces high concentrations of butyric acid that is are a beneficial microbial product in a mouse model of inflammatory bowel diseases [1]. Broccoli consumption decreases the relative abundance of Firmicutes and increases the abundance of Bacteroidates and Bacteroides in humans [2]. It also increases *Akkermansia muciniphila* abundance and reduces *Mucispirillum schaedleri* abundance [3]. Although broccoli exhibits health-beneficial effects by modulating gut microbiota, the effect of by-products from broccoli is rarely investigated. Broccoli by-products (BBP), which comprise the stems and leaves that are left after the broccoli head is harvested, is also of interest as a delivery matrix due to the abundant presence of nutrients consisting of 27.6% crude protein (N × 6.25), 5.5% fat and 54.9% carbohydrate [4]. The use of BPP helps to reduce food waste left in the field after harvest and is a route to add value to a underutilised product.

(-)-Epigallocatechin gallate (EGCG) is the most abundant bioactive compound in tea leaves with various biological activities, such as antioxidant capacity, anti-inflammatory and cardioprotective effects, as well as a regulatory effect on the gut microbiota [5,6,7,8]. A diet supplemented with EGCG (0.6%, *w*/*w*, 4 weeks) increased the starch and proteins in the feces of rats, suppressed the relative weight of abdominal adipose tissues, decreasing *Clostridium* spp. and increasing *Bacteroides* [5]. An in vitro study showed that EGCG treatment had no effect on the total population of bacteria but could significantly promote the growth of the *Lactobacillus-Enterococcus* group and *Bifidobaterium* spp. [6]. The metabolic fate of EGCG included consecutive ester hydrolysis, C-ring opening, A-ring fission, dehydroxylation and aliphatic chain shortening; overall, EGCG treatment stimulated beneficial bacteria [7]. EGCG attenuated non-alcoholic fatty liver disease via modulating gut microbiota composition and increasing the abundance of *Akkermansia* and *Parabacteroides* genera [8]. The potential of BBP as a delivery system for EGCG has been examined, and our previous studies showed that EGCG was protected by BBP during in vitro gastrointestinal digestion [9]. The co-delivery system of BBP and EGCG also exerted protection on tuna oil that contains long-chain omega-3 polyunsaturated fatty acids [4]. Omega-3 fatty acids decreased *Enterobacteria* while increasing *Bifidobacteria* and *Lachnospiraceae* in the gut, inducing the production of anti-inflammatory compounds and affecting the generation of short-chain fatty acids (SCFA) [10]. Dietary fibres, polyunsaturated fatty acids and polyphenols all have prebiotic potentials [11,12], and synergistic effects of two bioactives on the gut microbiota were observed [13].

To investigate the potential effect of BBPs on the gut microbiota and their health benefits, we examined the regulatory effects of BBP, EGCG-BBP, Tuna oil-BBP (TO-BBP) and Tuna oil-EGCG-BPP (TO-EGCG-BBP) formulations on the microbiota population and generation of SCFA in an in vitro anaerobic fermentation study, using human fecal inocula to mimic the microflora of the human large bowel. The selected metabolites of polyphenols after in vitro fermentation were identified by UPLC-MS. The aim of this study was to gain insights into the potential gut health benefits of the co-delivery system of EGCG and tuna oil using a broccoli matrix.

## 2. Results and Discussion

### 2.1. pH Value and Short-Chain Fatty Acids (SCFA) Changes

The pH values and the levels of three main short-chain fatty acids (acetate, butyrate, propionate) in all samples after 24 h fermentation in the fecal inoculum are shown in Table 1. The pH of the samples prior to fermentation was 6.8, while that of ferments after 24 h was between pH 4.2–6.8. The pH of ferments containing inulin was the lowest (pH 4.2), and the ferments containing BBP or BPP-TO were in the range of 5.2 to 5.4. All other samples had a pH higher than 6 (Table 1). In samples containing BPP, there was contribution resulting from the production of SCFA by the microbial fermentation of dietary carbohydrates and proteins. Bacterial conversion through reduction/hydrolysis of other dietary components (e.g., phytonutrients and polyunsaturated acids) into metabolites were also observed, contributing to the decrease in pH [14]. SCFA production was reduced by the incorporation of EGCG (Table 1). There are reciprocal interactions between polyphenols and gut microflora [15]. EGCG has been recently shown to modulate microflora in ovariectomized rats and high-fat diet-fed mice [16]. The effects of EGCG and broccoli polyphenols on modulating the microflora will also affect the concentration of bacterial metabolites and consequently the pH.

Levels of individual and total SCFA in ferments (Table 1) after 24 h fermentation were influenced by the type of substrate. The major SCFA produced were acetate, propionate and butyrate, with acetate being in greatest abundance. SCFA are physiologically important products of gut microbiota fermentation with multiple functions [17]. Acetate is a product of fermentation by many gut anaerobes and almost invariably is in the highest concentration among the SCFA in the gut lumen [18] and normally reaches peripheral tissues [19]. A high concentration of propionate in the colon has the potential for reducing cancer cell proliferation in the liver, where propionate is mainly taken up [20]. Butyrate is attractive because it has multiple benefits to the tissues of the colon. Butyrate serves as a histone deacetylase (HDAC) inhibitor, is the principal source of metabolic energy for colonocytes and is a G protein-coupled receptor (GPCR) activator [21]. The inclusion of inulin, BBP or TO-BBP significantly increased individual and total SCFA concentration relative to when no substrate was introduced or when cellulose, a known poor fermenter, was included (*p <* 0.05). The total SCFA, acetic acid and propionic acid of BBP and TO-BBP were 49.16, 31.78, 11.27 µmol/mL and 48.31, 29.75, 9.89 µmol/mL, respectively, which is higher than that of inulin, whereas inulin fermentation resulted in higher butyric acid production (9.62 µmol/mL) than BBP (5.87 µmol/mL) and TO-BBP (8.38 µmol/mL).

In a previous study, the in vitro fecal fermentation of broccoli fibre resulted in the production of acetate, propionate and butyrate in the molar proportions 46:10:7 [22]. After 24 h fermentation, no significant differences in individual and total SCFA production were observed between EGCG-BBP and EGCG-BBP- TO. EGCG-BBP and TO-EGCG-BBP had higher acetic acid (20.06 and 18.49 µmol/mL, respectively) and lower butyric acid (1.81 and 1.50 µmol/mL, respectively) relative to cellulose (13.03 µmol/mL for acetic acid and 3.16 µmol/mL for butyric acid). The SCFA production of TO was similar to that for cellulose. The valeric acid and branched-chain fatty acids (iso-butyric and iso-valeric acid) were only detected in minor amounts (<1.37 µmol/mL each). The distinct upregulation of SCFA by BBP and TO-BBP suggest these substrates have potentially beneficial effects on host metabolism.

Individual and total SCFA production was significantly inhibited by the presence of EGCG relative to that observed for cellulose (*p <* 0.05). Other studies reported that the total SCFA was enhanced significantly by EGCG during in vitro gut fermentation, and green tea polyphenols also induced the proliferation of certain beneficial bacteria and produced a relatively higher amount of SCFA [6,23]. However, the cecum of rats fed with a 0.6% EGCG diet produced less acetic and butyric acids, and EGCG had little effect on the production of propionic acid [5]. This suggests that the level of EGCG used in our study (15 mg/mL) is at a level sufficient to decrease SCFA production, an effect that is possibly related to concentration-dependent reciprocal interactions of polyphenols and microbiota.

### 2.2. Transformation of EGCG during In Vitro Microbiota Fermentation

Table 2 show that EGCG is transformed after 24 h of in vitro fermentation. Within the initial concentrations of EGCG in samples with introduced EGCG, only EGCG-BBP and BBP EGCG-TO were 15.00 mg/mL, 3.77 mg/mL and 3.03 mg/mL, respectively. After 24 h of in vitro microbiota fermentation, only 0.69 mg/mL and 1.02 mg/mL of EGCG were preserved in the samples containing EGCG-BBP and TO-EGCG-BBP, which corresponds to 18.3% and 33.7% not being transformed by fecal microflora. When EGCG was introduced alone, there was 7.83 mg/mL remaining as 52.2% EGCG was not transformed (Table 2). In our study, only gallic acid, EGC and GCG were detected. The concentrations of gallic acid, EGC and GCG in the sample containing EGCG were 0.17 mg/mL, 0.37 mg/mL and 1.31 mg/mL, which were much higher than those in the sample containing EGCG-BBP and TO-EGCG-BBP (Table 2).

For comparable loading amounts of EGCG in samples containing EGCG-BBP and TO-EGCG-BBP, it was found that TO protected EGC and GCG against further bacterial action, with 0.05 mg/mL EGC and 0.08 mg/mL GCG being retained, while no EGC and GCG were detected in the sample containing EGCG-BBP (Table 2). It is possible that the interactions of EGCG between the other components (proteins and fibre in BBP, tuna oil) altered the partitioning of EGCG, and consequently, the accessibility of EGCG to the microbiota. Gallic acid and EGC are the primary metabolites of EGCG due to the hydrolytic reaction during fermentation with intestinal bacteria [24], while GCG is the epimer of EGCG. Others have found that the biotransformation of EGCG during in vitro microbiota fermentation results in the generation of various metabolites such as EGC, gallic acid, pyrogallol, pyrocatechol, 5-(3’,4’,5’-Trihydroxyphenyl)-γ-valerolactone and so on [25,26]. Recently, a study revealed that EGCG was extensively catabolized with the obvious change of 14 metabolites by gut flora, including the metabolism of degalloylation, C-ring opening and A-ring fission [7]. EGCG was reported to decrease rapidly during the first 12 h of in vitro fermentation, forming EGC and gallic acid with the initial concentration of 0.1 mmol/L EGCG. EGCG went through further degradation from 24 h to 72 h [7]. This study indicates that these reactions are associated with microbial esterases, dehydroxylases and decarboxylases. However, there is limited information available on these enzymes [7]. Furthermore, only some of the metabolites reported by others have been found in our study, which was possibly due to the different conditions used and the effects of the high initial concentration of EGCG on the microbial enzymes. Microbiota-mediated biotransformation is also affected by the concentration of substrates [27].

### 2.3. Microbiota Population Changes

The majority of human fecal bacteria belong to the Bacteroidetes and Firmicutes phyla without substrate (Table 3). A decrease of Bacteroidetes was observed in the EGCG-containing samples, which is consistent with the previously reported results [7]. The relative abundances of Proteobacteria were 57.7%, 82.9% and 80.1% (EGCG, EGCG-BBP and TO-EGCG-BBP, respectively) at 24 h when compared to no substrate that had 8.39%. Proteobacteria is a marker for an unstable microbial community (dysbiosis) and a potential diagnostic criterion for disease [28]. High intake (1%) of EGCG in mice may induce the pro-inflammatory response [29]. Proteobacteria are able to target the inflamed niche by using nitrite produced from an inflammatory response [30]. The notable increase of Proteobacteria might be because of the high dose of EGCG as the gut microbiota required time to adapt [7].

The bacterial populations with close to or higher than 1% of total bacterial abundance at genus level are shown in Table 4. Health-promoting genera, including *Lactobacillus reuter* were highest in the BPP sample with the relative abundance of 9.80%, and *Bacillus coagulans* were higher in BBP and inulin with the relative abundance of 19.90% and 56.15%, respectively. An increase in abundance of these bacteria is able to inhibit pathogenic microbes and decrease the incidence of inflammatory disease [31,32]. The bacteria *Collinsella aerofaciens* has been implicated in the reduction of bloating in irritable bowel syndrome, and numbers were highest in the BPP sample (Table 4). BBP and TO-BBP increased the relative abundance of *Lachnospira* and *Veillonella dispar*. An increase in *Lachnospira* and *Veillonella* genera was also reported after functional omega-3 supplementation in humans [33,34]. These bacteria increased with BBP, indicating that BBP supplements might contribute to gut health. EGCG alone reduced the relative abundance of *Bacillus coagulans*, which was reported killed by the action of EGCG [35]. EGCG-BBP and TO-EGCG-BBP increased the relative abundance of *Gluconacetobacter*, *Klebsiella* and *Trabulsiella*, compared to no substrate and positive and negative controls as shown in Table 4. The increase in the relative abundance of *Gluconacetobacter* with EGCG is putatively related to EGCG metabolism. *Gluconacetobacter* has been reported to be isolated from kombucha [36,37]. The glucuronosyltransferase family from *Gluconacetobacter*, participates in polyphenol glucuronidation (hydrolysing its glycosidic bond). This could help polyphenols enter into enterohepatic circulation and result in a longer presence of polyphenols in the body, preventing diseases related to oxidative stress [38]. *Klebsiella* is often related to gut microbiome dysbiosis and can be multi-drug (antibiotic) resistant [39]. It also plays a role in polyphenol metabolism, indicating that the metabolism route for EGCG or *Klebsiella* is more resistant to EGCG [39]. This genus is generally considered to be a commensal opportunistic pathogen and has been shown to be highly resistant to polyphenolic compounds such as a methanolic extract of tea and EGCG, which are also able to metabolize EGCG [24,40,41]. The dominance of this species deviates from the in vivo situation and could be a consequence of the artificial nature of the gut model system in combination with the high dosage of polyphenols. TO enhanced the percentage of *Trabulsiella* compared to no substrate, whereas it was decreased in TO-BBP, which may be because of the high inhibition effect from BBP alone. Antibiotic-treated mice showed higher *Trabulsiella* than untreated groups, which indicate the high dose of EGCG used might act as an antibiotic component [42]. An increased relative abundance of *Trabulsiella* was also driven by a Citrobacter rodentium infection, which was inhibited by pomegranate peel extract [43]. However, there is no evidence that *Trabulsiella* is able to cause diarrhoea or intestinal infections, and the clinical significance of *Trabulsiella* is still unknown [44,45].

**Figure 1:**
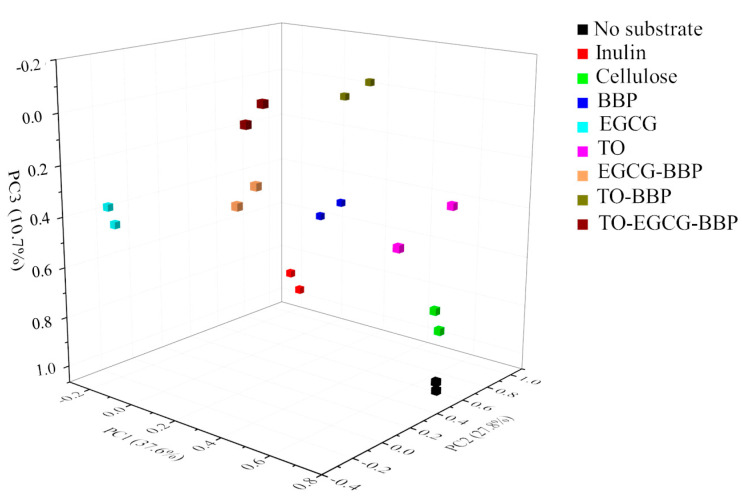
Principal component analysis of microbiota population changes at genus level at 24 h.

To compare the microbiota population of different substrates at genus level, the relative abundance of the bacterial genera from Table 4 was analyzed by principal component analysis (PCA), as shown in Figure 1. The first three principal components of PCA explained more than 76% of the variability, which was sufficient to clarify most of the differences among samples, indicating that the particular substrates had different effects on the colonic microbiota population.

### 2.4. Relationship between Microbiota Composition and SCFA Formation

**Figure 2:**
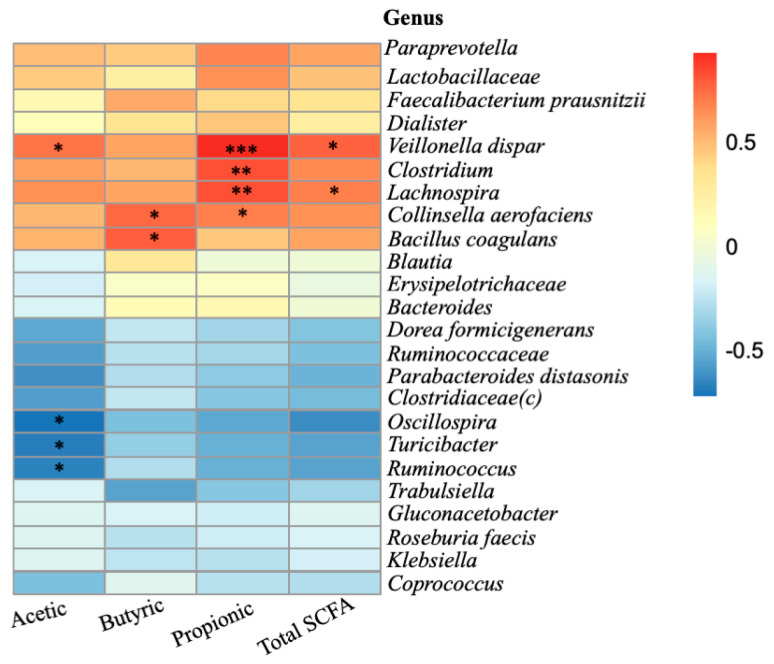
Pearson’s correlation between major microbiota and SCFA production at 24 h. * *p* < 0.05; ** *p* < 0.01; *** *p* < 0.005.

A heat map analysis of genera correlated with SCFA production is shown in Figure 2. *Clostridiaceae*, *Lachnospira*, *Collinsella aerofaciens* and *Veillonella dispar* were significantly associated with contributing to propionic acid formation (*p* < 0.05). It is likely that *Lactobacillus* produces lactic acid from BBP glucose, and then *Veillonella dispar* turns lactic acid into acetic acid and propionic acid [46]. *Lachnospira* members increased under the fermentation of soluble dietary fibre and may contribute to the production of SCFA [47]. Carbohydrate degradation by *Lachnospira* affects the growth of other bacteria, including other saccharolytic bacteria such as *Clostridium*, via cross-feeding [48]. The relative abundance of *Collinsella aerofaciens* and *Bacillus coagulans* was significantly and positively correlated with butyric acid production (*p <* 0.05). *Collinsella aerofaciens* is a novel butyrate producer isolated from a human gut [49], and *Bacillus coagulans* enhanced the production of butyric acid in cholesterol-rich foods [50]. *Oscillospira*, *Turicibacter* and *Ruminococcus* were negatively correlated with SCFA production (*p <* 0.05), reflecting the suppressive effect of these bacteria on SCFA production. *Oscillospira* and *Turicibacter* were decreased by the cooperation of *Prevotella*, *Lactobacillus* and *Streptococcus*, which may relate with multiple mechanisms such as secretion of host and microbial competition for nutrients [51].

There are several recognized limitations regarding our in vitro experimental approach. Firstly, all substrates were directly applied to human fecal inocula. Normally, in vivo, phenolic components will be absorbed in the small intestine and form conjugates. Our model assumes a high EGCG concentration which is mostly retained, and a low abundance of conjugates in the colon. Secondly, we have carried out fermentation over a 24 h period, but this may not be optimal for all formulations, as it is possible that the chemical structure and interaction within the different formulations may differ with time [13]. Thirdly, SCFA production and consumption by microbes would occur over time in our in vitro system. While this does occur in the in vivo setting, the colonic absorption of SCFA and other microbial metabolites that occurs in vivo is missing from the in vitro model and may significantly influence outcomes.

## 3. Materials and Methods

### 3.1. Material and Chemicals

Tuna oil (TO) was purchased from Nu-Mega Ingredients Pty Ltd. (Altona North, VIC Australia). EGCG powder (>95%) was provided by Sanfull Biological Technology Co., Ltd. (Hunan, China). Broccoli by-products (stems and leaves) were obtained from a local farm (Fresh Select, Werribee, VIC, Australia). EGCG, epigallocatechin (EGC), gallocatechin gallate (GCG), Gallic acid, standard (>99%) was purchased from Sigma-Aldrich (Castle Hill, NSW, Australia). Qiagen MagAttact Powder Microbiome Kit was purchased from Qiagen (Qiagen, Hilden, Germany).

### 3.2. Preparation of Formulations

Different formulated powders, including BBP, EGCG-BBP (25% EGCG:75% BBP, dry basis), TO-BBP (25% TO: 75% BBP, dry basis) and TO-EGCG-BBP (20% EGCG:20% TO:60% BBP, dry basis), were prepared and characterized as previously described (Shi et al., 2020). The obtained powders were stored at −20 °C until use.

### 3.3. In Vitro Gut Microbiota Fermentation

#### 3.3.1. Preparation of the Fermentation Medium

The growth medium was prepared according to our previous publication [13].

#### 3.3.2. Fresh Fecal Inoculum

Fresh fecal samples were collected and then pooled from three individual healthy human volunteers who were not on any dietary restrictions and had not taken antibiotics at least 3 months prior to donating. Fecal samples were transferred to an anaerobic chamber, and large solid particles were filtered. The equivalent amounts of faeces from each donor were mixed and diluted to 10% (*w*/*v*) with sterile anaerobic phosphate-buffered saline (PBS) (0.01 M, pH 7.2) and used as the fermentation starter. The slurry was homogenized and constantly stirred during inoculation into each fermentation test.

#### 3.3.3. Anaerobic Fermentation

Anaerobic fermentations were used to assess the effect of the introduced substrates (inulin, cellulose, BBP, EGCG, TO, BPP-EGCG, BPP-TO, BPP-EGCG-TO) on the fermentation characteristics and composition of the gut microbiota. For the control, no substrate was added. Anaerobic conditions were maintained throughout the set-up of fermentations using an anaerobic chamber (Bactron IV Anaerobic Chamber Sheldon Manufacturing Inc., Cornelius, OR, USA) to maximize the bacterial viability of the inoculum. Substrates at a concentration of 1.5% (*w*/*v*) in fermentation media were used in each test. Positive and negative control fermentations supplemented with inulin and cellulose at the same concentration were also included. Substrates were inoculated with 10% (*w*/*v*) of fresh fecal slurries. All the fermentations were incubated at 37 °C and gently mixed at 80 rpm. After the microbial fermentation (24 h), the end products were sampled for SCFA, selected polyphenol/metabolite and bacteria population analyses.

### 3.4. Phenolic Compounds Analysis

To analyze phenolic compounds, 4 mL of ferments was freeze-dried and extracted with 10 mL of 50% ethanol (*v*/*v*). The supernatants were filtrated through a 0.22 μm membrane and analyzed using a UHPLC-DAD-MS (Waters Corporation, Milford, MA, USA). The UHPLC and MS scan conditions were the same as those used previously (Zheng et al., 2018). An external standard method was used for the quantification of EGCG, gallic acid, EGC and GCG (λ = 280 nm).

### 3.5. Short Chain Fatty Acids (SCFA) Analysis

The short-chain fatty acids (SCFA) analysis, including chromatography specifications and calculation method, are as described previously [13], with slight modifications. Briefly, heptanoic acid (30 µL) as internal standard was added to 0.3 mL of each fermentation sample. Samples were mixed thoroughly and centrifuged at 2000× *g*, at 4 °C for 10 min. Then 30 µL of 1 M phosphoric acid was added to 300 µL of the supernatant. The fermentation samples were kept on ice to prevent SCFA volatilization throughout processing. Then 0.2 µL of filtered supernatant was injected into a gas chromatograph (model 7890A; Agilent Technologies, Santa Clara, CA, USA) equipped with a flame ionization detector and a capillary column (Zebron ZB-FFAP, 30 m × 0.53 mm × 1.0 µm, Phenomenex, Lane Cove, NSW, Australia).

### 3.6. DNA Extraction and 16 S Gene Sequencing

Aliquots (1 mL) of the fermentation samples were centrifuged at 14,000 rpm, at 4 °C for 5 min and the supernatants removed. For EGCG containing samples, sterile water (1 mL) was added to wash the microbial to remove free EGCG as EGCG binds to DNA and enzymes [52,53], which would result in low yield of DNA extraction and would inhibit PCR amplification. The procedure was repeated to remove the supernatant, and the precipitation was collected for DNA extraction. The DNA extraction of all precipitations was conducted following the protocol from the PowerMag^®^ Microbiome RNA/DNA Isolation Kit (27500-4-EP; MO BIO Laboratories, Inc., Carlsbad, CA, USA), optimized for epMotion^®^ platforms with slight modifications.

Briefly, 0.8 g of glass beads and 490 µL of pre-warmed PowerMag^®^ Microbiome Lysis Solution were added to faeces. Then the mixtures were homogenized (7000 rpm, 60 s) by a MagNAlyser, followed by centrifugation (10,000 rpm, 5 min) at 4 °C. The supernatant was collected, and 30 µL of Proteinase K > 600 mAU/mL (Qiagen, Hilden, Germany) was added. The sample was heated at 70 °C for 10 min, and 110 µL of PowerMag^®^ Inhibitor Removal Solution was added immediately after the heating. Samples were then incubated at −20 °C for 5 min and centrifuged at 14,000 rpm 15 for 5 min. The supernatant was recovered into a MO BIO 2 mL-deep well plate and 5 µL RNAse (10 mg/mL) was added to each sample. The remaining extraction procedure was carried out using the manufacturer’s protocol (epMotion-protocol-27500-V2.dws) optimized for epMotion^®^5075 (Eppendorf AG, Hamburg, Germany). DNA concentrations and purity were measured by Qubit and spectrophotometrically 20 (NanoDrop 1000 spectrophotometer, Thermo Fisher Scientific, Wilmington, DE, USA).

A broad assessment of microbial population changes was carried out by the PCR amplification of the 16S rRNA region of DNA extracted from the ferment samples. Sequencing was performed at the Australian Genome Research Facility. In brief, 300 bp sequencing was carried out on the V1–V3 region of the 16S rRNA region using an Illumina MiSeq. Paired-end reads were assembled by aligning the forward and reverse reads using PEAR (version 0.9.5). Primers were identified and trimmed. Trimmed sequences were processed using Quantitative Insights into Microbial Ecology (QIIME 1.8.4) (http://qiime.org/1.8.0/, access on 1 December 2021), USEARCH (version 30 8.0.1623) and UPARSE software(http://drive5.com/uparse/, access on 1 December 2021). Using USEARCH tools, sequences were quality filtered, full-length duplicate sequences were removed and sorted by abundance. Singletons or unique reads in the data set were discarded. Sequences were clustered, followed by chimera filtered using the “rdp_gold” database as a reference. To obtain the number of reads in each operational taxonomic unit (OTUs), reads were mapped back to OTUs with a minimum identity of 97%. Taxonomy was assigned using QIIME.

### 3.7. Statistical Analysis

Polyphenol/metabolite analysis and SCFA measurements were conducted in triplicates. DNA extraction and 16 S gene sequencing were two replicates. Data are expressed as a mean ± standard deviation (SD). One-way ANOVA (Origin) assessed the mean differences between groups. Statistical significance was accepted at *p* < 0.05.

## 4. Conclusions

The results of the current study, obtained using an in vitro fermentation system containing human stool, reports the effects of EGCG, TO or a combination co-delivered by BBP on EGCG change, SCFA production and microbiota community changes. EGCG inhibited the generation of SCFA, suggesting that the concentration of EGCG in dietary supplements may influence its prebiotic properties in vivo. Although EGCG-BBP and TO-EGCG-BBP increased the proportion of *Trabulsiella* during fermentation, the clinical function of *Trabulsiella* is still unknown. BBP and TO-BBP fermentation stimulated the production of butyric acid, one of the most physiologically beneficial products. This, together with a capacity to increase the population of beneficial microbes such as *Lactobacillus reuteri*, indicates these two formulations could potentially be used as prebiotics for human consumption. In the future, combining in vitro mechanistic insight with in vivo studies will be required for a more accurate assessment of the deep relationship and mechanisms in the formulation–microbiota–host triangle.

## Figures and Tables

**Table 1:** The pH value and SCFAs concentration (µmol/mL ± STD) after 24 h batch fermentations ^1^.

Substrates	pH at 24 h ^2^	Total SCFA ^3^ (µmol/mL)		Acetic Acid Amount (µmol/mL)		Propionic Acid Amount (µmol/mL)		Butyric Acid Amount (µmol/mL)	
		T = 0 h	T = 24 h	T = 0 h	T = 24 h	T = 0 h	T = 24 h	T = 0 h	T = 24 h
No substrate	6.8	1.72 ± 0.21 ^a^	21.99 ± 2.07 ^d^	1.21 ± 0.13 ^a^	13.99 ± 1.42 ^f^	0.23 ± 0.03 ^a^	2.53 ± 0.21 ^d^	0.22 ± 0.03 ^b^	2.83 ± 0.21 ^e^
Inulin	4.2	2.21 ± 0.64 ^a^	42.80 ± 2.56 ^b^	1.54 ± 0.42 ^a^	26.41 ± 1.50 ^c^	0.31 ± 0.10 ^a^	6.37 ± 0.53 ^c^	0.30 ± 0.09 ^ab^	9.62 ± 0.49 ^a^
Cellulose	6.4	2.21 ± 1.22 ^a^	21.35 ± 1.47 ^d^	1.52 ± 0.79 ^a^	13.06 ± 0.98 ^f^	0.32 ± 0.22 ^a^	2.63 ± 0.16 ^d^	0.30 ± 0.19 ^ab^	3.16 ± 0.19 ^de^
BBP	5.2	2.52 ± 0.69 ^a^	49.16 ± 2.18 ^a^	1.45 ± 0.20 ^a^	31.78 ± 1.35 ^a^	0.40 ± 0.18 ^a^	11.27 ± 0.46 ^a^	0.43 ± 0.20 ^a^	5.87 ± 0.35 ^c^
TO	6.5	1.56 ± 0.25 ^b^	25.48 ± 0.87 ^c^	1.03 ± 0.14 ^a^	15.98 ± 0.46 ^e^	0.21 ± 0.04 ^a^	3.26 ± 0.20 ^d^	0.21 ± 0.04 ^b^	3.64 ± 0.10 ^d^
EGCG	6.5	1.40 ± 0.12 ^b^	4.48 ± 0.69 ^e^	1.01 ± 0.06 ^a^	3.26 ± 0.41 ^g^	0.18 ± 0.01 ^b^	0.59 ± 0.09 ^e^	0.17 ± 0.01 ^b^	0.41 ± 0.11 ^g^
EGCG-BBP	6.1	1.57 ± 0.06 ^b^	25.20 ± 1.63 ^c^	1.13 ± 0.04 ^a^	20.06 ± 0.64 ^d^	0.19 ± 0.00 ^b^	3.14 ± 0.67 ^d^	0.18 ± 0.00 ^b^	1.81 ± 0.25 ^f^
TO-BBP	5.4	1.78 ± 0.05 ^a^	48.31 ± 1.22 ^a^	1.33 ± 0.03 ^a^	29.75 ± 0.56 ^b^	0.21 ± 0.01 ^b^	9.89 ± 0.35 ^b^	0.19 ± 0.00 ^b^	8.38 ± 0.28 ^b^
TO-EGCG-BBP	6.1	1.56 ± 0.03 ^b^	22.71 ± 1.44 ^c,d^	1.15 ± 0.02 ^a^	18.49 ± 0.70 ^d^	0.18 ± 0.00 ^b^	2.62 ± 0.56 ^d^	0.17 ± 0.00 ^b^	1.50 ± 0.16 ^f^

^1^ Different letters (a–g) indicate a significant difference in the same column at the same time (*p <* 0.05). ^2^ The starting pH of samples are around 6.8. ^3^ Total SCFA means the total acid of acetic, propionic, butyric, iso-butyric, valeric, iso-valeric and caproic.

**Table 2:** The biotransformation of EGCG after 24 h in vitro microbiota fermentation (mg/mL).

Sample	Initial Concentration of EGCG	After 24 h Fermentation			
		EGCG	Gallic Acid	EGC	GCG
EGCG	15.00 ± 0.12 ^a^ (100%)	7.83 ± 1.01 ^a^ (52.2%)	0.17 ± 0.02 ^a^	0.37 ± 0.01 ^a^	1.31 ± 0.23 ^a^
EGCG-BBP	3.77 ± 0.04 ^b^ (100%)	0.69 ± 0.05 ^b^ (18.3%)	0.05 ± 0.01 ^b^	NA	NA
TO-EGCG-BBP	3.03 ± 0.02 ^c^ (100%)	1.02 ± 0.14 ^b^ (33.7%)	0.05 ± 0.01 ^b^	0.05 ± 0.01 ^b^	0.08 ± 0.00 ^b^

Different letters (a–c) indicate a significant difference in the same column (*p <* 0.05). The values in brackets are the remaining of EGCG: the remaining of EGCG (%) = EGCG amount at 24 h/initial EGCG amount × 100%.

**Table 3:** Effects of formulations (BBP with EGCG, TO or a combination of EGCG and TO) on shifts in gut microflora at phylum level in vitro colonic fermentation model at 24 h.

	No Substrate	Inulin	Cellulose	BBP	EGCG	TO	EGCG-BBP	TO-BBP	TO-EGCG-BBP
Firmicutes	75.3 ± 0.5	91.1 ± 1.2	74.9 ± 0.9	88.5 ± 4.2	33.1 ± 0.5	64.9 ± 9.0	13.5 ± 0.1	82.9 ± 3.3	13.7 ± 0.3
Bacteroidetes	14.9 ± 0.7	6.5 ± 0.8	13.7 ± 0.5	9.4 ± 3.9	8.3 ± 1.2	14.6 ± 0.6	3.3 ± 0.3	14.9 ± 3.1	6.1 ± 0.4
Proteobacteria	8.4 ± 0.9	1.4 ± 0.3	9.7 ± 0.3	0.9 ± 0.0	57.7 ± 1.6	19.6 ± 9.8	82.9 ± 0.3	1.2 ± 0.0	80.1 ± 0.2
Others	1.4 ± 0.3	1.1 ± 0.1	1.7 ± 0.1	1.2 ± 0.4	0.9 ± 0.1	1.0 ± 0.3	0.3 ± 0.1	1.0 ± 0.2	0.2 ± 0.0

**Table 4:** Effects of formulations (BBP with EGCG, TO or a combination of EGCG and TO) on gut microflora in vitro colonic fermentation model at 24 h.

	No Substrate	Inulin	Cellulose	BBP	EGCG	TO	EGCG-BBP	TO-BBP	TO-EGCG-BBP
*Coriobacteriaceae* *(Collinsella aerofaciens)*	0.45 ± 0.0007	0.90 ± 0.0000	0.75 ± 0.0007	0.95 ± 0.0035	0.25 ± 0.0007	0.40 ± 0.0014	0.15 ± 0.0007	0.70 ± 0.0014	0.05 ± 0.0007
*Bacteroidaceae* *(Bacteroides)*	10.30 ± 0.0042	5.35 ± 0.0064	10.00 ± 0.0028	7.70 ± 0.0339	6.20 ± 0.0113	10.70 ± 0.0042	2.80 ± 0.0014	12.60 ± 0.0311	5.25 ± 0.0035
*Porphyromonadaceae (Parabacteroides distasonis)*	3.50 ± 0.0042	0.80 ± 0.0014	2.45 ± 0.0021	0.80 ± 0.0014	0.65 ± 0.0007	2.90 ± 0.0014	0.20 ± 0.0000	0.95 ± 0.0007	0.25 ± 0.0007
*Paraprevotellaceae (Paraprevotella)*	0.35 ± 0.0021	0.15 ± 0.0007	0.55 ± 0.0007	0.75 ± 0.0021	0.70 ± 0.0000	0.20 ± 0.0000	0.15 ± 0.0007	1.05 ± 0.0007	0.30 ± 0.0000
*Bacillaceae* *(Bacillus coagulans)*	2.45 ± 0.0007	56.15 ± 0.0615	2.45 ± 0.0007	19.90 ± 0.0184	2.40 ± 0.0000	2.50 ± 0.0085	0.45 ± 0.0007	7.60 ± 0.0071	0.40 ± 0.0014
*Lactobacillaceae* *(Lactobacillus reuteri)*	1.30 ± 0.0028	1.45 ± 0.0049	1.55 ± 0.0007	9.80 ± 0.0113	1.95 ± 0.0064	1.95 ± 0.0120	0.20 ± 0.0000	1.00 ± 0.0042	0.15 ± 0.0007
*Turicibacteraceae (Turicibacter)*	0.95 ± 0.0007	0.45 ± 0.0007	1.30 ± 0.0014	0.50 ± 0.0000	0.90 ± 0.0014	0.95 ± 0.0007	0.30 ± 0.0000	0.35 ± 0.0007	0.40 ± 0.0000
*Clostridiaceae* *(Clostridium)*	0.90 ± 0.0170	1.30 ± 0.0042	3.40 ± 0.0028	14.10 ± 0.0325	0.70 ± 0.0014	4.05 ± 0.0035	0.35 ± 0.0007	17.45 ± 0.0049	0.45 ± 0.0007
*Clostridiaceae (c)*	4.80 ± 0.0007	0.65 ± 0.0007	1.80 ± 0.0014	0.55 ± 0.0007	0.70 ± 0.0028	1.40 ± 0.0014	0.20 ± 0.0000	0.50 ± 0.0000	0.25 ± 0.0007
*Lachnospiraceae* *(Blautia)*	2.15 ± 0.0028	8.30 ± 0.0057	8.65 ± 0.0092	3.95 ± 0.0007	1.65 ± 0.0007	6.35 ± 0.0049	0.90 ± 0.0014	4.25 ± 0.0049	0.75 ± 0.0007
*Lachnospiraceae (Coprococcus)*	4.95 ± 0.0028	1.15 ± 0.0035	2.35 ± 0.0021	1.20 ± 0.0014	1.95 ± 0.0007	1.55 ± 0.0007	0.90 ± 0.0014	1.55 ± 0.0007	1.10 ± 0.0000
*Lachnospiraceae* *(Dorea formicigenerans)*	7.70 ± 0.0042	2.75 ± 0.0049	12.75 ± 0.0021	3.40 ± 0.0028	1.50 ± 0.0014	10.05 ± 0.0106	0.70 ± 0.0014	4.15 ± 0.0021	0.75 ± 0.0007
*Lachnospiraceae* *(Lachnospira)*	2.00 ± 0.0007	0.50 ± 0.0014	0.90 ± 0.0000	4.50 ± 0.0028	0.50 ± 0.0000	0.95 ± 0.0007	0.35 ± 0.0007	7.75 ± 0.0318	0.45 ± 0.0007
*Lachnospiraceae* *(Roseburia faecis)*	13.00 ± 0.0007	0.05 ± 0.0007	0.70 ± 0.0000	0.30 ± 0.0014	1.90 ± 0.0014	0.50 ± 0.0000	0.80 ± 0.0014	1.00 ± 0.0042	1.15 ± 0.0007
*Ruminococcaceae*	0.95 ± 0.0085	0.95 ± 0.0007	4.65 ± 0.0021	1.80 ± 0.0014	2.05 ± 0.0007	3.90 ± 0.0014	0.70 ± 0.0014	2.30 ± 0.0014	0.80 ± 0.0014
*Ruminococcaceae (Fecalibacterium prausnitzii)*	0.45 ± 0.0035	5.55 ± 0.0049	6.05 ± 0.0021	5.10 ± 0.0057	7.25 ± 0.0021	5.20 ± 0.0099	1.85 ± 0.0007	8.40 ± 0.0071	1.75 ± 0.0007
*Ruminococcaceae (Oscillospira)*	4.70 ± 0.0042	0.40 ± 0.0014	3.55 ± 0.0007	0.45 ± 0.0007	0.90 ± 0.0000	3.50 ± 0.0028	0.35 ± 0.0007	0.55 ± 0.0007	0.35 ± 0.0007
*Ruminococcaceae (Ruminococcus)*	5.45 ± 0.0014	4.05 ± 0.0021	9.10 ± 0.0071	1.80 ± 0.0014	2.45 ± 0.0007	8.55 ± 0.0092	1.50 ± 0.0000	2.60 ± 0.0014	1.75 ± 0.0007
*Veillonellaceae* *(Dialister)*	4.10 ± 0.0014	0.85 ± 0.0007	1.30 ± 0.0014	1.35 ± 0.0035	1.00 ± 0.0000	1.30 ± 0.0028	0.70 ± 0.0028	1.50 ± 0.0014	0.30 ± 0.0000
*Veillonellaceae* *(Veillonella dispar)*	9.30 ± 0.0007	1.35 ± 0.0021	1.35 ± 0.0007	11.85 ± 0.0148	0.15 ± 0.0007	1.00 ± 0.0028	NA	10.55 ± 0.0134	NA
*Erysipelotrichaceae*	0.45 ± 0.0007	0.30 ± 0.0014	1.30 ± 0.0000	0.60 ± 0.0014	0.35 ± 0.0007	1.00 ± 0.0014	0.20 ± 0.0000	1.40 ± 0.0014	0.30 ± 0.0014
*Enterobacteriaceae (Gluconacetobacter)*	0.45 ± 0.0000	NA	0.10 ± 0.0000	NA	16.35 ± 0.0078	0.40 ± 0.0028	3.35 ± 0.0134	NA	2.65 ± 0.0106
*Enterobacteriaceae* *(Klebsiella)*	0.45 ± 0.0000	0.05 ± 0.0007	0.30 ± 0.0000	NA	39.55 ± 0.0064	1.50 ± 0.0085	14.35 ± 0.0559	0.05 ± 0.0007	12.95 ± 0.0530
*Enterobacteriaceae (Trabulsiella)*	0.45 ± 0.0099	0.85 ± 0.0035	8.20 ± 0.0028	0.40 ± 0.0000	0.80 ± 0.0014	16.30 ± 0.0877	64.95 ± 0.0658	0.65 ± 0.0007	64.30 ± 0.0622

## Data Availability

The data presented in this study are available in the paper.

## Sample Availability

Samples of the compounds are available from the authors.
